# Vegetation-mediated assembly of rhizosphere fungal communities and their ecological drivers in constructed wetland systems

**DOI:** 10.3389/fmicb.2025.1686352

**Published:** 2026-01-05

**Authors:** Nan Deng, Yuxin Tian, Qingan Song, Yandong Niu, Fengfeng Ma

**Affiliations:** 1 Hunan Academy of Forestry, Changsha, Hunan, China; 2 Dongting Lake National Positioning Observation and Research Station of Wetland Ecosystem of Hunan Province, Yueyang, China; 3 Hunan Cili Forest Ecosystem State Research Station, Hunan, China; 4 International Technological Cooperation Base for Ecosystem Management and Sustainable Utilization of Water Resources in Dongting Lake Basin, Changsha, China

**Keywords:** constructed wetlands, rhizosphere fungal communities, community assembly, dispersal limitation, homogeneous selection

## Abstract

**Introduction:**

Constructed wetlands (CWs) are widely applied for restoring degraded wetland ecosystems and enhancing pollutant removal. Rhizosphere fungal communities play a crucial role in these ecosystems by mediating nutrient cycling and pollutant degradation.

**Methods:**

This study examined the diversity patterns and assembly mechanisms of rhizosphere fungal communities across seven different CW types with distinct vegetation compositions within a farmland-to-wetland conversion system.

**Results:**

We identified 6,243 operational taxonomic units (OTUs), with sequencing coverage exceeding 98% across all vegetation types. Significant differences in phylogenetic diversity were observed between vegetation types. The dominant fungal classes were Sordariomycetes and Glomeromycetes, with ternary plot analysis indicating that different plant species selectively enriched distinct fungal lineages. Soil physicochemical properties, including pH, salinity, organic matter content, total potassium, and available phosphorus, showed significant variation across the different vegetation types. These soil factors were strongly correlated with the composition and diversity of rhizosphere fungal communities. Beta nearest taxon index (β MNTD) analysis indicated that both deterministic and stochastic processes contributed to community assembly, with stochastic processes exerting a stronger influence. The iCAMP model revealed that dispersal limitation was the primary factor influencing community assembly (57.13%), followed by homogeneous selection (20.53%) and drift processes (19.91%). Mantel tests showed that key environmental factors at key as soil nutrient levels, enzyme activities, and plant biomass was significantly associated with specific fungal lineages and community structure.

**Discussion:**

These findings provide new insights into ecological processes that govern rhizosphere fungal community assembly in CWs, thereby advancing the evaluation of wetland restoration effectiveness and informing microbial management strategies to optimize ecosystem services and pollutant removal in CW systems.

## 1 Introduction

Wetlands rank as some of the Earth’s most productive and ecologically valuable ecosystems, offering essential services like treating wastewater, reducing floods, supplying wildlife habitats, and capturing carbon (Shiau et al., 2019; Messer et al., 2017). Characterized by widespread anaerobic soil environments, these ecosystems play a vital role in worldwide biogeochemical processes, especially in the cycling of carbon and nitrogen (Zhang et al., 2025). Despite their ecological importance, wetland ecosystems face unprecedented threats from anthropogenic activities. In the last century, human activities have altered or degraded more than half of the Earth’s wetlands (Sica et al., 2016), making them one of the most threatened ecosystems globally (Junk et al., 2013). In response to wetland loss and degradation, constructed wetlands (CWs) have emerged as a promising nature-based solution. These engineered systems replicate natural wetland functions by utilizing physical, chemical, and biological processes to treat wastewater and restore ecological functions (Lin et al., 2025; Zhang et al., 2025). The growing recognition of CWs as efficient and cost-effective technologies has led to their widespread adoption globally (Zhang et al., 2021). However, the success of CWs depends on understanding and optimizing the complex interactions between vegetation, microbial communities, and environmental factors.

The efficacy of CWs is fundamentally driven by intricate relationships between vegetation and associated microorganisms. Plants facilitate microbial colonization through root surface attachment and biofilm formation, thereby creating diverse microenvironments that support varied ecological processes (Fu et al., 2023; Shi et al., 2023). Different plant species possess distinct functional traits that influence rhizosphere fungal communities through multiple pathways. Root system architecture-including fibrous versus taproot systems, root depth, and branching patterns-directly influences fungal colonization by providing varied physical attachment sites and creating heterogeneous microhabitats with differential oxygen availability and moisture retention (Bonfante and Genre, 2010; Prada-Salcedo et al., 2022). Root exudate profiles, comprising organic acids (e.g., citric acid, malic acid), sugars, amino acids, and phenolic compounds, selectively recruit fungal taxa through carbon source provisioning and chemical signaling, thereby driving deterministic selection processes (Wu et al., 2024; Zhalnina et al., 2018). Plant growth forms and biomass allocation patterns further modulate fungal assembly by altering litter input, soil physical structure, and nutrient cycling rates (Wardle et al., 2004; Ping et al., 2019). High-biomass species typically enhance carbon availability and stabilize soil aggregates, potentially strengthening environmental filtering effects, while deep-rooted plants modify vertical soil stratification and facilitate fungal dispersal through extensive hyphal networks (Freschet et al., 2021). These plant trait-mediated mechanisms collectively determine whether fungal community assembly is governed by deterministic processes (e.g., environmental selection driven by trait-induced niche differentiation) or stochastic processes (e.g., dispersal limitation influenced by root architecture and spatial distribution patterns) (Dini-Andreote et al., 2015; Zhou and Ning, 2017). Thus, plant species differ in root architecture, exudate release, and habitat formation, collectively fostering diverse fungal communities with varied metabolic capacities (Rani et al., 2024). Microbial communities, particularly fungi, play pivotal roles in wetland ecosystem functioning through their involvement in organic matter decomposition, nutrient cycling, soil quality enhancement, and pollutant transformation (Hyde et al., 2019; Lin et al., 2022; Xun et al., 2025). Fungal community diversity serves as a valuable indicator for assessing soil nutrient status, environmental quality, and overall ecosystem health and stability (Rodriguez et al., 2019, Adamo et al., 2021). Research has demonstrated that wetland degradation leads to significant declines in both bacterial and fungal community diversity (Lin et al., 2023). The composition and species diversity of soil fungal communities vary considerably across different stages of wetland degradation, reflecting the sensitivity of these communities to environmental changes (Li et al., 2023; Jiang et al., 2024).

Despite the acknowledged importance of plant-microbe interactions in wetland ecosystems, significant knowledge gaps remain regarding rhizosphere fungal communities in restored wetlands. Current research on rhizosphere fungal community assembly processes under different vegetation types is particularly limited, hindering our understanding of how restoration strategies can be optimized through targeted vegetation selection. However, it remains unclear whether high-biomass plant species enhance deterministic selection through increased carbon inputs and environmental modification, or whether plants with extensive fibrous root systems promote stochastic assembly by facilitating fungal dispersal across soil microhabitats (Stegen et al., 2012; Tripathi et al., 2018). In addition, how plant traits interact with environmental gradients (e.g., nutrient availability and soil moisture) to jointly regulate the balance between deterministic and stochastic processes in fungal community assembly remains poorly understood (Martiny et al., 2006; Chase, 2010). Moreover, the mechanisms underlying fungal community assembly and the key environmental factors shaping community structure in CWs are still largely unknown. This study focuses on a constructed wetland system established through farmland-to-wetland conversion, representing a common restoration approach. The study area features multiple vegetation types and management patterns, providing an ideal natural laboratory for investigating plant-fungal interactions. Understanding the co-occurrence patterns and community assembly mechanisms of fungal communities is essential for evaluating the effectiveness of artificial restoration efforts and gaining insights into ecological processes that govern wetland ecosystem functioning. The objectives of this study were to: (i) test whether rhizosphere fungal communities differ significantly across vegetation types in CWs; and (ii) determine which assembly processes and environmental drivers shape these communities.

## 2 Materials and methods

### 2.1 Study area and sample collection

The study was conducted in the demonstration area of returning farmland to wetland, Xiangjia Village, Yongzhou City, Hunan Province, China. This area is located in the river wetlands in the middle reaches of the Xiangjiang River Basin (Geographic coordinates of center point: 111°45’58.0824”E, 26°34’36.6204”N). The study site was previously agricultural land in a densely populated area, where wetland pollution was relatively serious due to perennial rice cultivation and domestic sewage discharge from surrounding residents.

**FIGURE 1:**
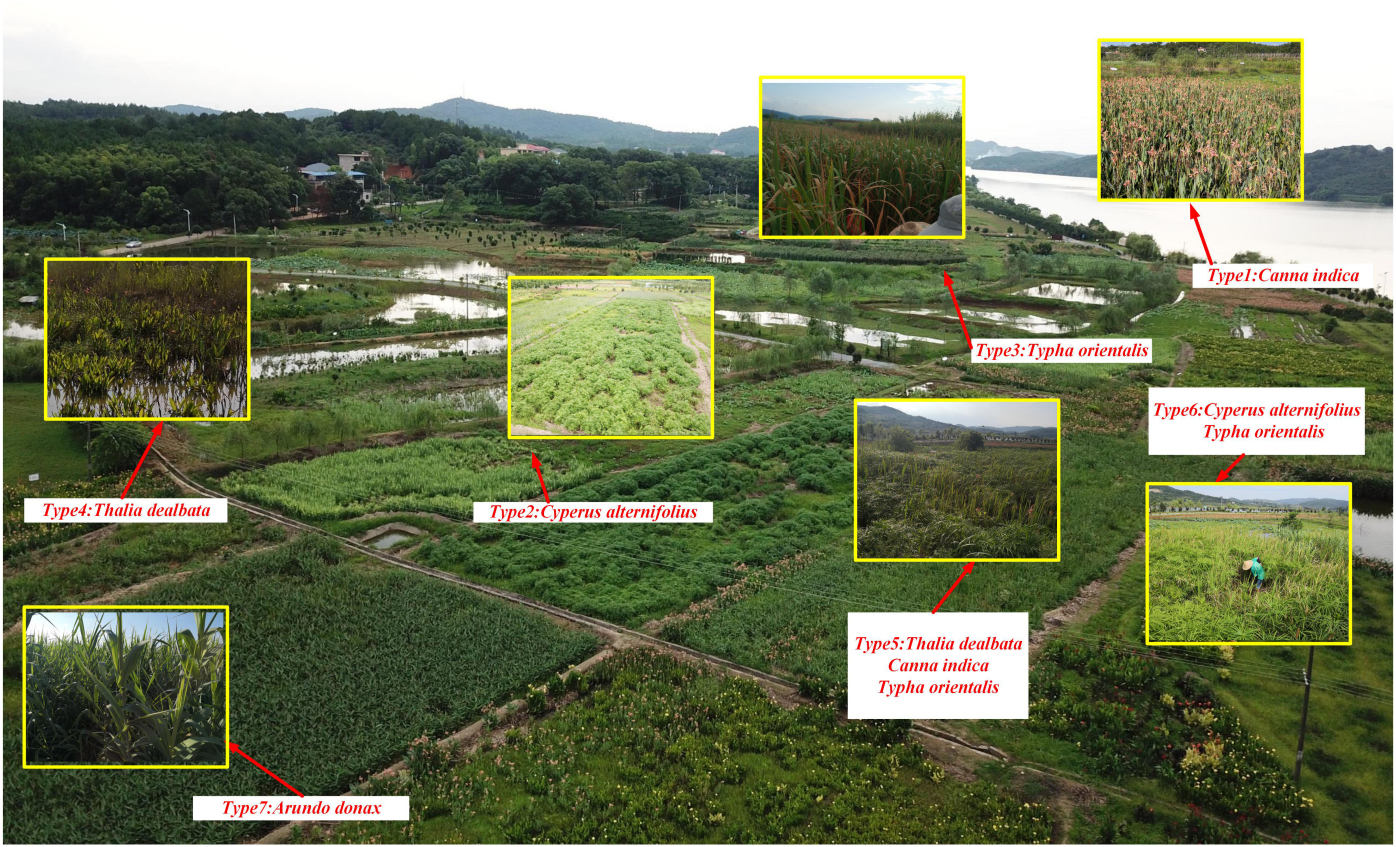
Layout of seven experimental plots. Each plot (300–800 m^2^) was established on former farmland following the natural topographic gradient to preserve hydrological flow patterns.

To restore these degraded wetlands, a series of CWs with different plant compositions were established. Seven experimental plots were designed to represent different wetland plant community types (Table 1). The CWs were designed and installed based on the natural topographic gradient of the original farmland, which was carefully preserved during construction to maintain the natural hydrological flow patterns. Each plot covered 300–800 m^2^ and was physically separated from adjacent plots by raised banks (40–50 cm height) to prevent cross-contamination and ensure independent treatment conditions (Figure 1). No additional groundworks beyond the installation of separating banks were conducted, as the natural gradient of the original farmland provided adequate drainage and water distribution across the experimental area. All experimental plots were constructed simultaneously 3 years prior to sampling, allowing sufficient time for plant establishment and ecosystem stabilization. The experimental design included five monoculture treatments (types 1–4 and 7) and two polyculture treatments (types 5–6), enabling comparison of plant diversity effects on wetland ecosystem functions. The water supply for all experimental plots originated from the collection and centralized discharge of surrounding environmental waters, including natural precipitation runoff and controlled drainage from the adjacent areas. The wetlands operate under a continuous flow-through system without regular flooding or artificial inundation events. All sampling sites were strategically positioned in the central discharge zone to ensure uniform water distribution and similar hydraulic conditions across treatments. Water quality parameters were monitored across all plots to confirm no significant differences in baseline water chemistry, ensuring that observed differences between treatments could be attributed to plant community composition rather than varying water quality conditions. To minimize potential environmental confounding factors, all experimental plots were positioned within the same general area with similar exposure to wind and solar radiation. The compact spatial arrangement of the plots within the central discharge zone ensured that microclimate variations, including differences in sun exposure and wind patterns, were minimized and unlikely to significantly influence the measured ecosystem parameters between treatments.

**TABLE 1:** Types of CWs with plant composition.

Type ID	Plant composition	Cover contribution (%)
1	*Canna indica* L.	90
2	*Cyperus alternifolius* L.	95
3	*Typha orientalis* Presl	95
4	*Thalia dealbata* Fraser	70
5	*Thalia dealbata*	30
	*Canna indica*	30
	*Typha orientalis*	30
6	*Cyperus alternifolius*	50
7	*Typha orientalis*	40
	*Arundo donax* L.	95

Three biological replicates were collected from each of the seven wetland types, resulting in a total of 21 samples (*n* = 3 per treatment). Within each plot, three small plots (1 m^2^) separated by at least 10 m were selected to ensure spatial independence and representative sampling within each treatment. The growth status of plant such as height and coverage was measured, and then all plants in each small plot were removed to collect samples. To obtain the rhizosphere soil, which was defined as soil particles tightly adhering to the root surface after gentle manual shaking, the topsoil (0–5 cm) was removed and the whole root system was dug out (20–40 cm), the plants were gently shaken, and then the tightly bonded soil that remained attached to the root surface was collected. All collected rhizosphere soil samples were kept separate by sampling location (no pooling across the three biological replicates within each plot) and immediately transported to the laboratory in 4C°. Each soil sample (filtered by 2 mm mesh) was then divided into two parts: one part was stored at 4C° for physicochemical analysis, and the other was used for DNA extraction.

### 2.2 Physicochemical analyses

The plants were divided into aboveground and underground parts and measured separately. Total biomass, aboveground biomass, and belowground biomass (all in kg/m^2^) were calculated. A total of 15 soil and 4 leaf physicochemical properties were measured. Soil chemical properties were determined as follows: give soil to extractant ratio for pH method; organic matter by the potassium dichromate method; total salinity by electrical conductance; total nitrogen by potassium dichromate-sulfuric acid digestion; total phosphorus by sulfuric acid-perchloric acid digestion followed by Mo-Sb colorimetry; total potassium by NaOH fusion and flame photometry; available nitrogen by the Conway method; available phosphorus by NaHCO3 extraction with Mo-Sb colorimetry; and available potassium by NH_4_OAc extraction with flame photometry. Soil enzyme activities were measured using the following methods: catalase by permanganate titration; sucrase by the 3,5-dinitrosalicylic acid method; urease by indophenol blue colorimetry; phosphatase by sodium diphenyl phosphate colorimetry; and protease by the casein hydrolysis method. Soil cadmium content was determined by atomic fluorescence spectrometry.

Soil physical properties were measured using samples collected with a cutting ring. The following parameters were determined: non-capillary porosity, capillary porosity, total porosity, bulk density, minimum water-holding capacity, capillary water holding capacity, and maximum water holding capacity (Philip, 1957).

### 2.3 DNA extraction, sequencing, and bioinformatics analyses

Total microbial DNA was extracted using the FastDNA Spin kit (MP Biomedicals, Santa Ana, CA) according to the manufacturer’s instructions. The fungal ITS1 region was amplified using the primer pair ITS1F (5′-CTTGGTCATTTAGAGGAAGTAA-3′) and ITS2 (5′-GCTGCGTTCTTCATCGATGC-3′) for each sample. High-throughput sequencing was performed on an Illumina MiSeq platform (Illumina, Inc., San Diego, CA) with paired-end (2 × 300 bp) reads.

The obtained FASTQ files were subjected to quality control using Trimmomatic (v0.36) and PEAR (v0.9.6). Trimmomatic applied a sliding window of 50 bp with an average quality threshold of Q20 and a minimum read length of 120 bp, while PEAR was used to remove sequences containing ambiguous bases (N). Subsequently, FLASH (v1.2.10) and PEAR were employed to merge paired-end reads based on overlapping regions, with a minimum overlap length of 10 bp and a maximum mismatch rate of 0.1, generating high-quality FASTA sequences. Chimeric sequences were identified and removed using the UCHIME algorithm implemented in Vsearch (v2.7.1) with both reference-based and *de novo* detection modes, and low-quality or short reads were discarded.

High-quality sequences were further processed in QIIME (v1.8.0) and QIIME2 (version 2019) following the standard workflow (Bolyen et al., 2019).^1^ Sequences were clustered into operational taxonomic units (OTUs) at a 97% similarity threshold using the UPARSE (v7.0.1) algorithm. Taxonomic annotation was performed against the UNITE fungal ITS reference database (release 7.2). Sequence alignment was conducted using MAFFT, and a phylogenetic tree was constructed with FastTree.

### 2.4 Fungal community structure and ecological process analysis

Alpha diversity indices including Chao1, goods coverage, observed species, phylogenetic diversity (PD whole tree), Shannon, and Simpson indices were calculated using the “vegan” package in R after rarefying all samples to an equal sequencing depth of 30,000 reads per sample. Multiple comparative analysis (Tukey HSD test) was performed to detect significant differences among different plant types at the 95% family-wise confidence level. Beta diversity was analyzed using Bray-Curtis dissimilarity matrices. Beta diversity components were partitioned into replacement, richness difference, and similarity components using ternary plots to illustrate the relative contributions of these components to overall community dissimilarity. Ternary plots were constructed to visualize class-level composition differences of rhizosphere fungal communities among different plant types, with each point representing the relative abundance of fungal classes across three selected plant types.

To assess the main effects of environmental factors on fungal community structure, permutational multivariate analysis of variance (PERMANOVA; vegan:adonis2) was conducted on Hellinger-transformed OTU data with 999 permutations. Prior to analysis, variance inflation factors (VIF) were calculated for all candidate environmental variables, and variables with VIF > 10 were iteratively removed until multicollinearity was minimized, resulting in 16 environmental factors for subsequent analyses. *P*-values from multiple hypothesis testing were corrected using the false discovery rate (FDR) method with Benjamini-Hochberg correction. Non-metric multidimensional scaling (NMDS) ordination was performed using the “vegan” package, and significant environmental factors (*p* ≤ 0.05) were fitted onto the NMDS ordination space using the envfit function with 999 permutations to obtain vector directions, *R*^2^-values, and significance levels. Distance-based redundancy analysis (dbRDA; vegan:capscale) was employed to quantify the proportion of variance explained by environmental factors, with overall and per-axis tests reported alongside adjusted R^2^ values. To further partition the contributions of environmental factors, hierarchical variance partitioning was performed using the rdacca.hp package (Lai et al., 2022).

To characterize the phylogenetic community composition, we quantified the beta mean nearest taxon distance (betaMNTD) among pairwise samples, which measures phylogenetic turnover based on the mean phylogenetic distance between the nearest taxon in one community and taxa in another community (Stegen et al., 2012). The relationships among beta diversity (Bray-Curtis distance), environmental distance, and betaMNTD were examined using Mantel and partial Mantel tests. Environmental distance was calculated as Euclidean distance based on the 16 selected environmental variables after standardization. Mantel tests were performed with 9,999 permutations to assess statistical significance. The matrices of β-diversity were divided into decomposed, replacement and richness difference that represented the triple values of replacement, richness difference and similarity corresponding to a point in a triangular graph (Legendre, 2014), and ternary plots were constructed to visualize the relative contributions of these components to overall community dissimilarity. All analysis was conducted using the R package of “picante” (Kembel et al., 2010), “vegan” (Jari et al., 2019), and “ape” (Paradis and Schliep, 2019).

To reveal the rhizosphere fungal community assembly processes, iCAMP model was employed, using the “iCAMP” package in R (Ning et al., 2020). The first step involved phylogenetic binning, which divided the fungal community into 31 taxonomic bins based on phylogenetic relationships. Five assembly processes were then examined based on the bin-based null model analysis. The five assembly processes were divided into deterministic [heterogeneous selection (HeS) and homogeneous selection (HoS)] and stochastic processes [dispersal limitation (DL), homogeneous dispersal (HD) and drift and other processes (DR)]. For bins associated with non-neutral processes (bins with HoS values exceeding 0.16), environmental drivers were identified through Mantel tests between the betaMNTD-corrected dissimilarities of each bin and environmental variables. Additionally, we computed the partial Mantel correlations between the phylogenetic bins.

## Results

### 3.1 Soil and plant trait differentiation among different plant types

**FIGURE 2:**
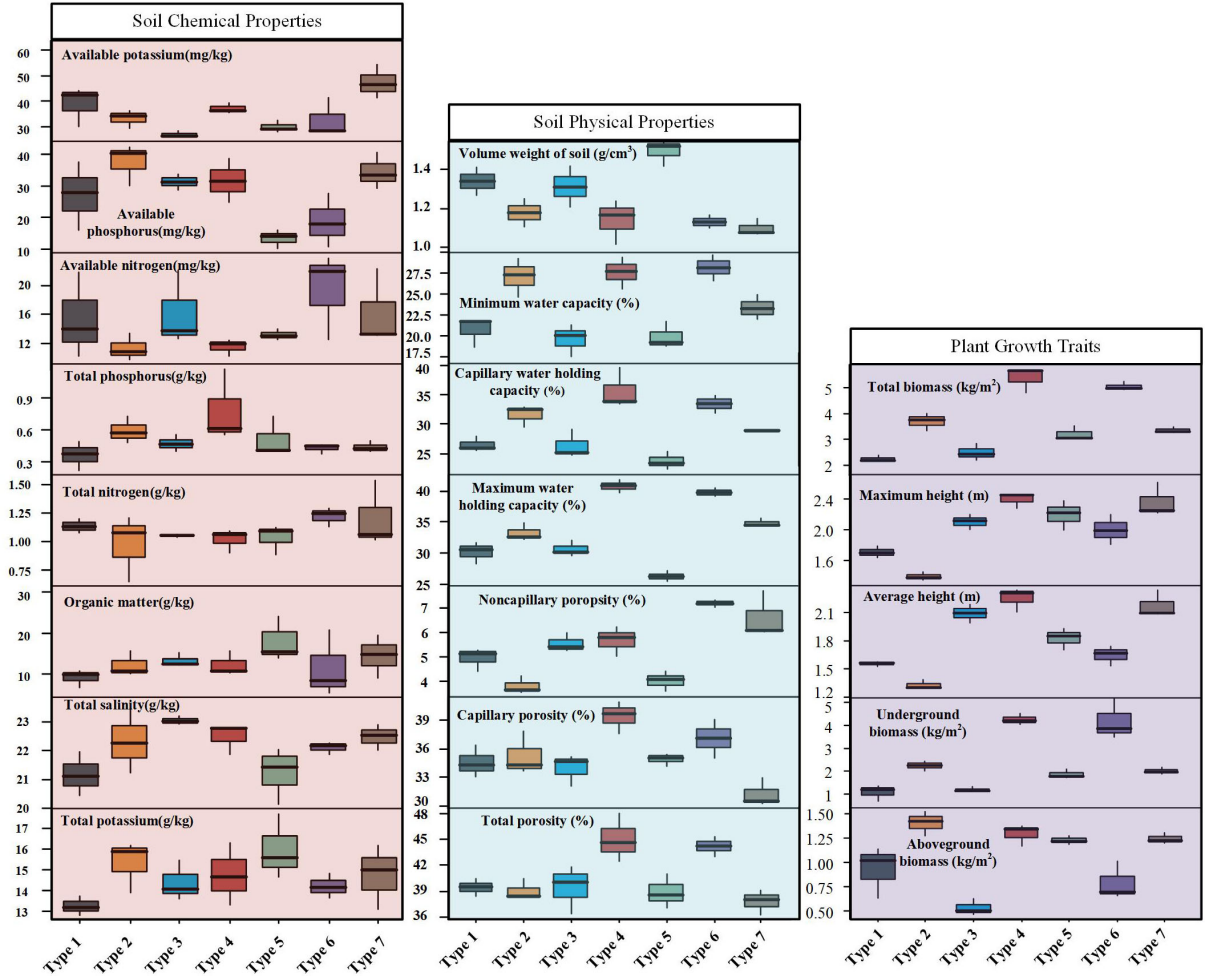
Boxplot comparisons of 20 measured indicators across seven constructed wetland plant community types, including soil chemical properties (8 indicators), soil physical properties (7 indicators), and plant growth traits (5 indicators).

A total of 20 indicators were measured across the seven wetland plant community types, including 8 soil chemical properties, 7 soil physical properties, and 5 plant growth traits (Figure 2). The results revealed varying degrees of differentiation among community types across different indicator categories. Soil chemical properties showed limited variation, with only two out of eight indicators exhibiting significant differences among community types. Available potassium differed significantly between type 3 and type 7, while available phosphorus showed more widespread variation across five community pairs (type 3 vs. type 6, type 1 vs. type 6, type 1 vs. type 7, type 2 vs. type 3, and type 1 vs. type 2; *p* < 0.05). The remaining six chemical indicators showed no significant differences. In contrast, soil physical properties demonstrated extensive variation among community types. Six out of seven physical indicators showed significant differences, with water retention and porosity characteristics being particularly variable. Volume weight of soil varied significantly across six community pairs, while minimum water capacity and capillary water holding capacity differed in nine and eight pairs, respectively. Soil porosity measurements showed the most extensive variation, with non-capillary porosity exhibiting significant differences in 12 community pairs, followed by total porosity (seven pairs) and capillary porosity (five pairs; all *p* < 0.05). Plant growth traits exhibited the most comprehensive differentiation among community types. All five growth indicators showed significant differences, with total biomass displaying variation across most community pairs (significant in all except five pairs). Plant height measurements revealed distinct patterns, with maximum height differing significantly in nine pairs and average height in 11 pairs. Biomass allocation also varied considerably, with underground and aboveground biomass showing significant differences in 11 and 10 community pairs, respectively (*p* < 0.05). These results indicate that different plant community compositions create distinct microenvironmental conditions and exhibit contrasting growth strategies in the constructed wetland system.

**FIGURE 3:**
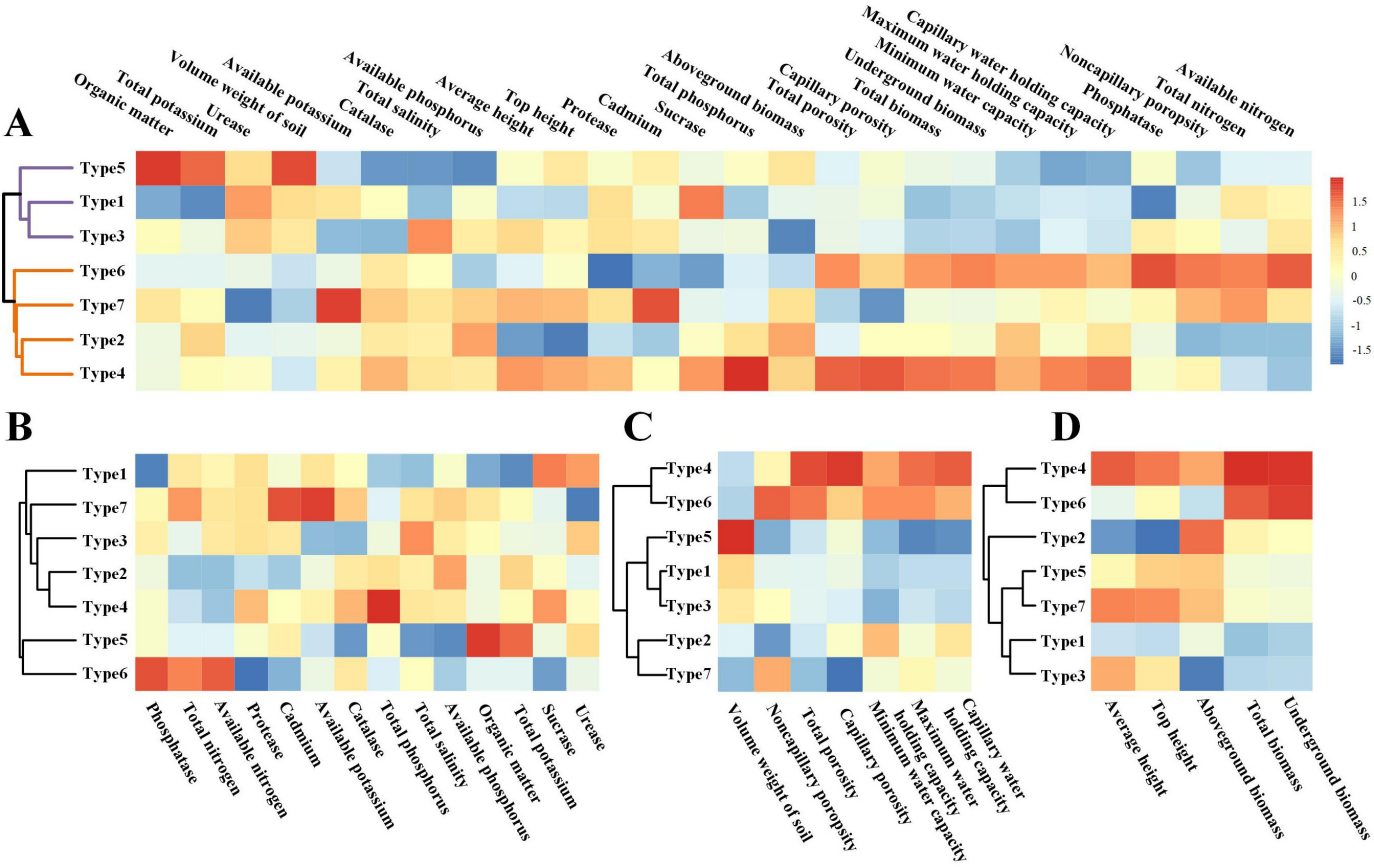
Heatmap and hierarchical cluster analyses of soil and plant growth indicators across seven constructed wetland plant community types. **(A)** The clustering based on all 20 measured indicators, while **(B–D)** display clustering patterns based on soil chemical properties (8 indicators), soil physical properties (7 indicators), and plant growth traits (5 indicators), respectively. All data were standardized prior to heatmap generation and cluster analysis.

Cluster analysis and heatmap visualization of standardized data revealed clear differentiation patterns of soil and plant traits among the seven plant types (Figure 3A). The comprehensive cluster analysis, integrating all measured indicators, divided the communities into two major groups (Figure 3A). Group I comprised type 1, type 3, and type 5, whereas Group II included type 2, type 4, type 6, and type 7 (*A. donax*). Heatmap analysis further highlighted the contrasting trait profiles between groups. Group I (types 1, 3, 5) was characterized by significantly lower biomass indicators (aboveground, underground, and total biomass), weaker soil physical properties (including porosity and water-holding capacities), and reduced nutrient-related measures (phosphatase activity, total nitrogen, and available nitrogen). In contrast, Group II displayed generally higher values for these indicators, reflecting stronger growth performance and more favorable soil conditions.

Separate cluster analyses based on different indicator categories revealed distinct grouping patterns. Soil chemical indicators produced two main groups: type 5 and type 6 showed the highest similarity, while the remaining five types (type 1, 2, 3, 4, and 7) clustered together (Figure 3B). Soil physical properties formed a three-cluster structure, with type 2 and type 7 in one cluster, type 1, 3, and 5 in a second, and type 4 and 6 in a third (Figure 3C). Plant growth traits displayed a binary pattern, with TYPE 4 and 6 clustering together and the other five types (type 1, 2, 3, 5, and 7) grouped into a single cluster (Figure 3D). Overall, the cluster analysis results of different indicator types showed certain differences, indicating that soil chemical properties, soil physical properties, and plant growth traits have relatively independent response patterns among different plant community types, while comprehensive analysis better reflects the overall differentiation characteristics among different plant types.

### 3.2 Alpha diversity index of rhizosphere fungi

**FIGURE 4:**
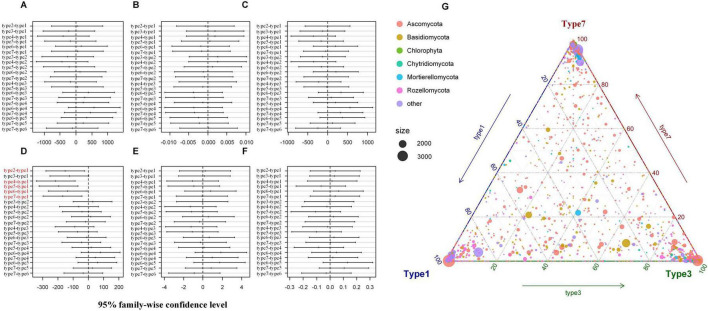
Alpha diversity indices and community composition differences of rhizosphere fungi among different plant types. **(A–F)** Present multiple comparative analyses (Tukey HSD, 95% family-wise confidence level) of six alpha diversity indices (Chao1, goods coverage, observed species, PD whole tree, Shannon, and Simpson). **(G)** Displays a ternary plot representing the class-level composition differences of rhizosphere fungal communities among plant types 1, 3, and 7. Each point in the ternary plot indicates the relative abundance of a fungal class across the three plant types.

High-throughput sequencing yielded a total of 6,243 OTUs across all rhizosphere types. Sequencing coverage exceeded 98% for each type, indicating that the data reliably captured the fungal community composition. Alpha diversity indices showed no significant differences among rhizosphere types, with the exception of the PD whole tree index. This metric revealed that type 1 had significantly higher phylogenetic diversity than types 2, 4, 5, 6, and 7 (Figures 4A–F) (*p* < 0.05).

The fungal community composition across the seven rhizosphere types shows significant variations in class abundance. Sordariomycetes and Glomeromycetes are the most abundant, indicating their key roles in nutrient cycling and symbiosis, especially in type 1. Mortierellomycetes also shows high abundance in multiple types, particularly in type 5 and type 7, highlighting its potential importance in soil health. In contrast, Pucciniomycetes and Archaeosporomycetes are less abundant and appear in only a few types. Rozellomycota_cls_Incertae_sedis is highly abundant in type 2, suggesting type-specific variations. Additionally, Leotiomycetes and Eurotiomycetes are consistently present across several types, particularly in type 1, type 5, and type 6, indicating their involvement in plant interactions in different rhizosphere types. From the ternary plot depicting fungal class compositions in rhizosphere communities of type 1, type 3, and type 7 plants (Figure 4G), clear clustering of different fungal classes around the three vertices was observed. This indicates that each plant type tends to selectively enrich certain fungal groups. For instance, types 1 and 3 were associated with high-abundance groups such as Ascomycota and Rozellomycota, which dominated their rhizosphere fungal communities. Ascomycota emerged as the most prevalent phylum, distributed throughout the ternary space but concentrated particularly at the plot corners, suggesting its ubiquity across plant types alongside specific genus-level preferences. Meanwhile, Basidiomycota, Chytridiomycota, and Rozellomycota were more localized toward individual vertices, indicating selective enrichment by specific host plants. These findings support the idea that host identity plays a key role in shaping rhizosphere fungal communities.

**FIGURE 5:**
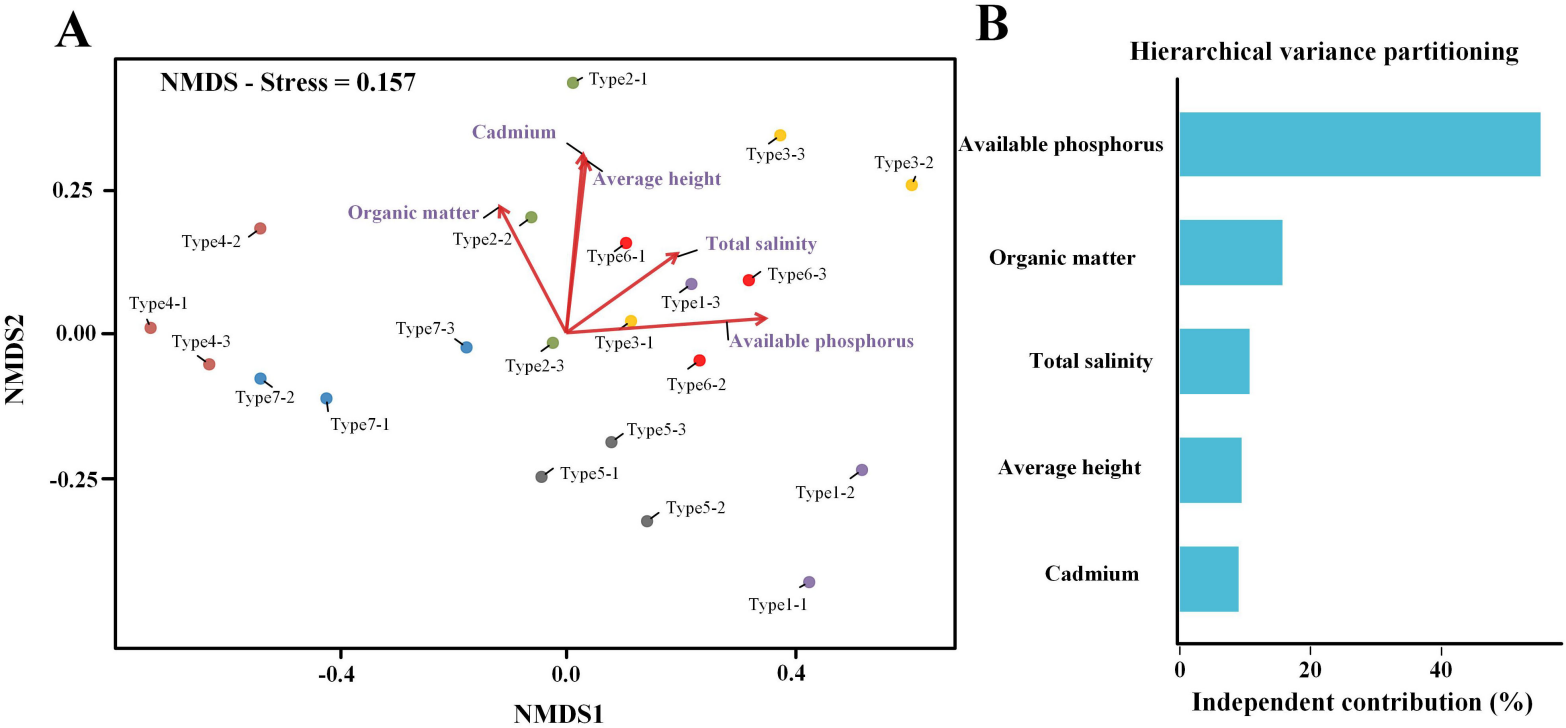
Fungal community structure and environmental drivers across different plant types in wetlands. **(A)** Non-metric multidimensional scaling (NMDS) ordination of fungal communities based on Hellinger-transformed data. Different colors represent different plant types (type 1–7). Each point represents a sample, with three replicates per plant type. Arrows indicate the direction and strength of environmental variables significantly correlated with community structure (envfit, *p* < 0.05). Arrow length is proportional to the correlation strength. **(B)** Hierarchical variance partitioning using the rdacca.hp package quantifying the independent contribution of each environmental factor to the variation in fungal community composition. Bar height indicates the percentage of total explained variation uniquely attributed to each factor.

PERMANOVA analysis revealed significant differences in fungal community composition among different plant types (*R*^2^ = 0.85, *p* = 0.02). NMDS ordination analysis further revealed the spatial distribution patterns of community structure (Figure 5), with a stress value of 0.157 indicating good ordination quality, tight clustering of replicate samples from each site, and distinct clustering of different plant types in two-dimensional ordination space. Environmental factor fitting analysis (envfit) identified five environmental variables significantly correlated with fungal community structure (*p* < 0.05): total salinity, organic matter content, available phosphorus, cadmium content, and average height, where plant types 4, 7, and 2 were mainly distributed in areas with lower available phosphorus content, while plant types 1, 3, 5, and 6 were distributed in areas with higher available phosphorus content (Figure 5A). Hierarchical variance partitioning results showed that Available phosphorus (55.2%) and Organic matter (15.6%) were the major contributors to the explained variation in community composition, followed by average height (9.4%), Cadmium (8.9%), and salinity (10.7%), indicating that phosphorus nutrition is a key driving factor shaping wetland fungal community structure (Figure 5B).

### 3.3 Fungal community assembly processes

**FIGURE 6:**
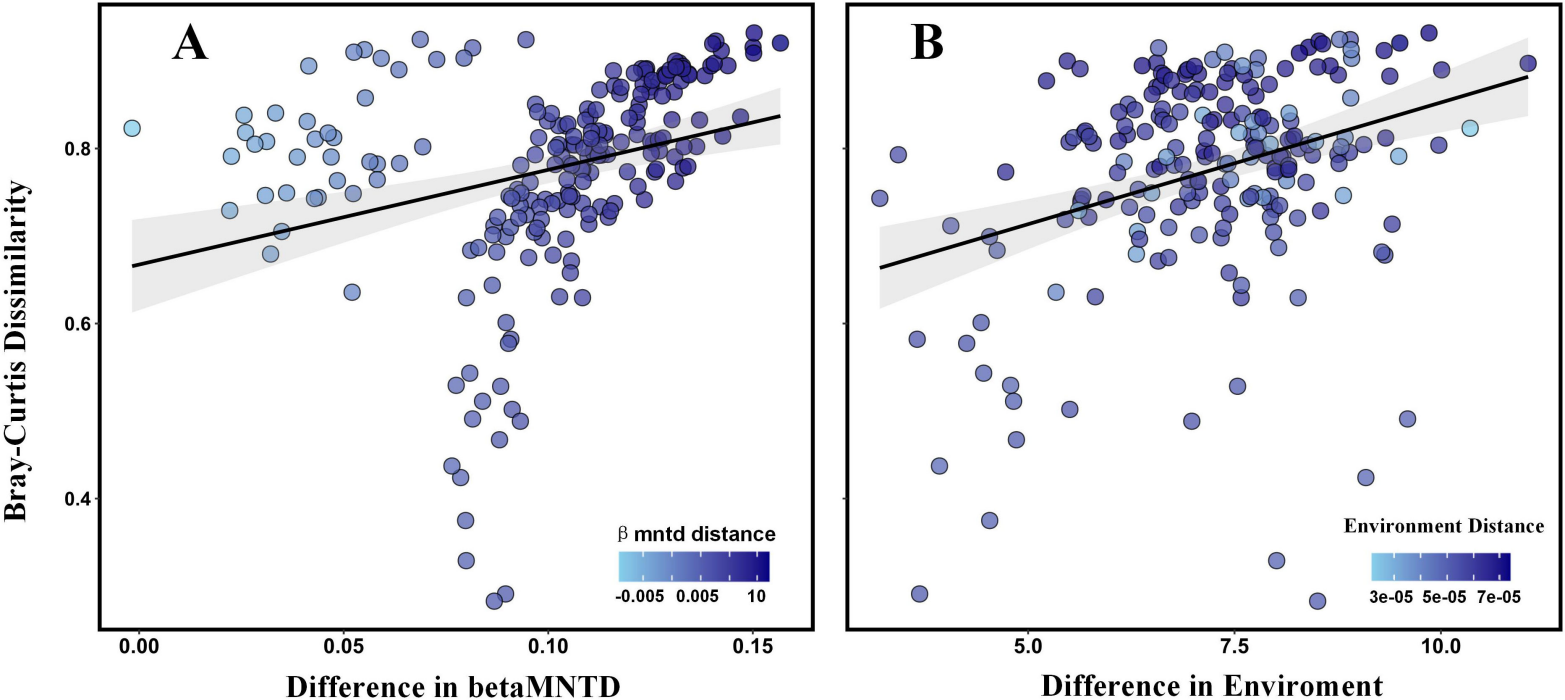
Variation in β diversity along betaMNTD **(A)** and environment distance **(B)**. Each point represents a pairwise comparison among rhizosphere communities. The fitted regression line with 95% confidence interval (shaded area) illustrates the general trend.

Bray-Curtis dissimilarity analyses (Figures 6A,B) reveal clear positive relationships between fungal β diversity and both phylogenetic dissimilarity (betaMNTD) and environmental distance. In Figure 6A, Bray-Curtis dissimilarity increases with greater differences in betaMNTD, suggesting that phylogenetically distinct communities exhibit more divergent compositions. Similarly, Figure 6B shows that as environmental conditions diverge, fungal community structure becomes increasingly dissimilar, underscoring the role of environmental filtering. These graphical observations are quantitatively supported by the Mantel and partial Mantel test results (Table 2). A significant positive correlation was found between β diversity and environmental distance (*r* = 0.3264, *p* = 0.001), which remained robust even after controlling for phylogenetic distance (*r* = 0.3352, *p* = 0.001). Likewise, β diversity also correlated significantly with betaMNTD (*r* = 0.2842, *p* = 0.001), and this relationship persisted after accounting for environmental distance (*r* = 0.2946, *p* = 0.001). These findings confirm that both environmental heterogeneity and phylogenetic divergence independently shape fungal community composition.

**TABLE 2:** Mantel and partial mantel test between β diversity and environmental distance/BetaMNTD.

Statistical indicator	Environmental distance	Environmental distance eliminate BetaMNTD	BetaMNTD	BetaMNTD eliminate environmental distance
Statistic r	0.3264	0.3352	0.2842	0.2946
Significance	0.001***	0.001***	0.001***	0.001***

“***” represent the significant level of 0.001, 0.01, and 0.05.

### 3.4 Rhizosphere fungal community composition and distribution patterns

**FIGURE 7:**
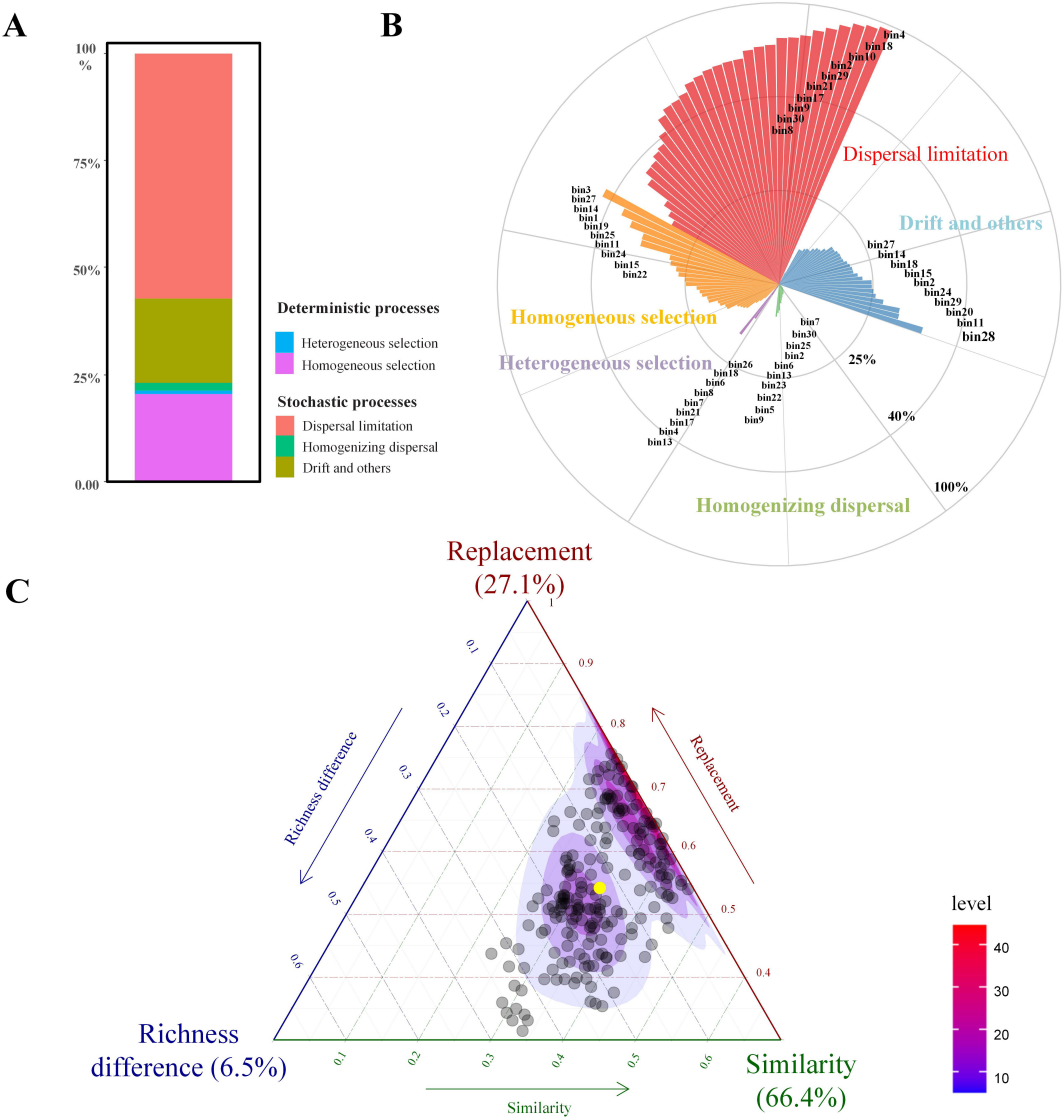
Integrated analysis of β-diversity components and ecological assembly processes in the soil fungal community. **(A)** Displays the relative contributions of deterministic processes (e.g., heterogeneous selection, homogeneous selection) and stochastic processes (e.g., dispersal limitation, homogeneous dispersal, and undominated processes) to community assembly. **(B)** Displays the process contributions partitioned within 31 taxonomic bins, showing the dominant ecological drivers at the lineage level. Only the top 10 bins for each assembly process are listed. **(C)** Displays ternary plots of β-diversity, partitioned into replacement, richness difference, and similarity components, illustrating how different pairwise community dissimilarities are distributed among these three elements.

The iCAMP analysis of ecological processes revealed that dispersal limitation (DL) was the predominant driver of soil fungal community assembly (Figure 7A). At the whole-community level, DL accounted for 57.13% of the total contribution, followed by homogeneous selection (HoS) and drift and other processes (DR), with contributions of 20.53 and 19.91%, respectively. In contrast, heterogeneous selection (HeS) and homogeneous dispersal (HD) showed minimal influence, contributing only 0.62 and 1.81%, respectively. These results suggest limited environmental heterogeneity and restricted long-distance dispersal within the study system. Further partitioning across 31 taxonomic bins showed that 27 bins (87.1%) were predominantly governed by DL, while only four bins (bin3, bin14, bin27, and bin28) were dominated by HoS. No bins were found to be primarily shaped by HeS, HD, or DR. Among all bins, the average contribution of DL exceeded 55%, with several bins (e.g., bin4, bin10, bin18) reaching over 70%, indicating strong spatial structuring. In contrast, HoS-dominated bins exhibited highly variable values, reflecting stronger environmental filtering only within specific fungal lineages (Figure 7B).

The ternary plot (Figure 7C) illustrates the partitioning of β diversity into replacement, richness difference, and similarity components across soil fungal communities. The results indicate that similarity accounted for the largest proportion (66.4%), suggesting that most sample pairs shared a substantial number of fungal taxa, resulting in relatively low β diversity. Replacement contributed 27.1%, indicating that in a notable fraction of sample pairs, community differences arose from species turnover rather than richness imbalance. This aligns with the observed importance of dispersal limitation in the community assembly process. In contrast, richness difference explained only 6.5% of the dissimilarity, implying that differences in species richness played a minimal role in shaping fungal community variation across sites. These results suggest that the soil fungal communities in this system are characterized by high compositional similarity and that species turnover, more than richness differences, contributes to observed dissimilarity.

**FIGURE 8:**
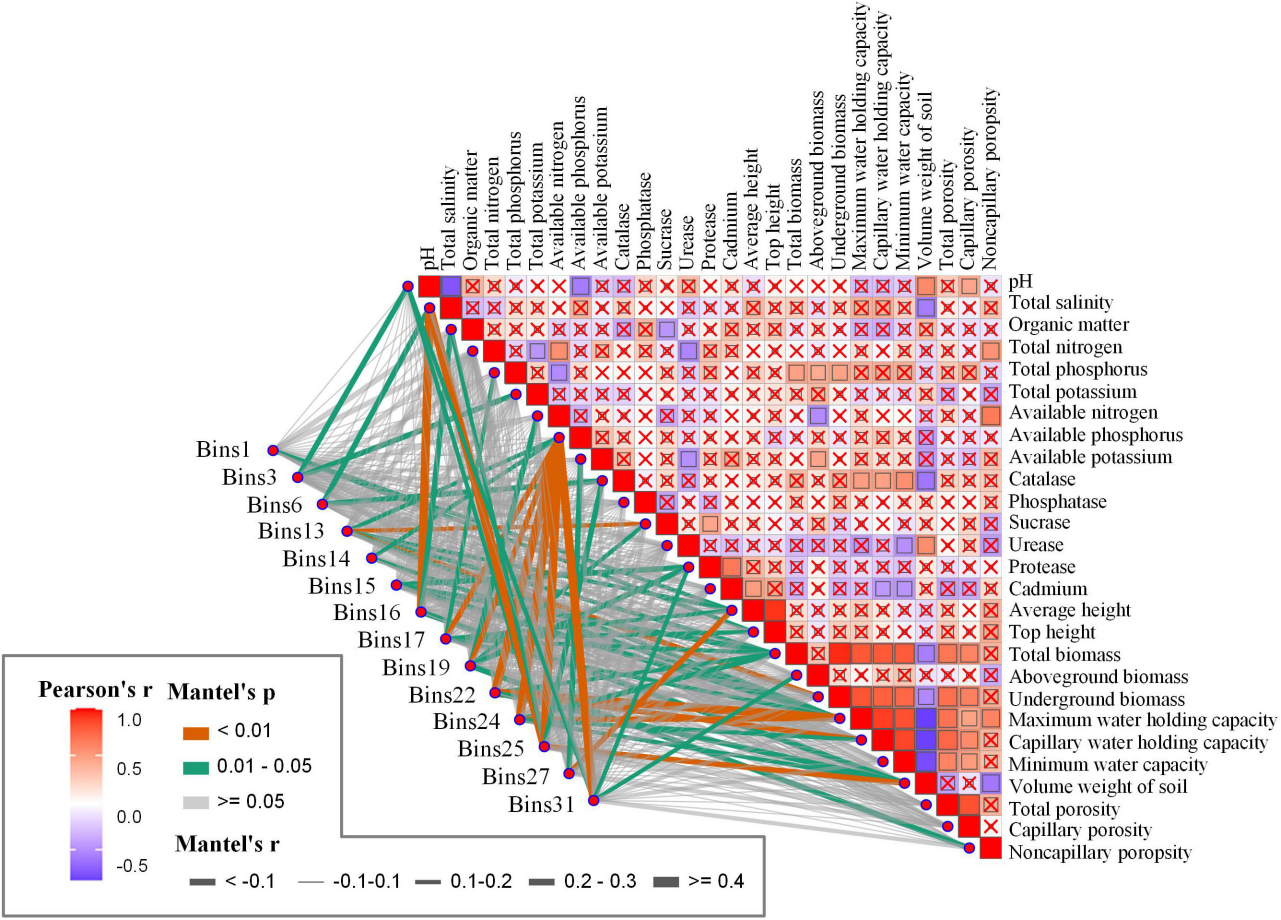
Environmental drivers associated with the composition of fungal taxonomic bins in rhizosphere communities. Pairwise associations between environmental variables and community dissimilarities of bins dominated by non-neutral processes (HoS > 0.16) were assessed using Mantel tests with betaMNTD-corrected dissimilarities. Edge width represents the Mantel’s r statistic, and edge color denotes statistical significance based on 9,999 permutations. The color gradient in the matrix indicates Spearman’s correlation coefficients between variables, while “x” marks non-significant correlations. Significant associations highlight environmental factors such as pH, total potassium, available phosphorus, salinity, organic matter, and plant traits as key drivers of community differentiation across bins.

To further investigate the environmental drivers of bins associated with non-neutral processes (i.e., bins with HoS values exceeding 0.16, totaling 14 bins) in rhizosphere communities, we performed Mantel tests between the betaMNTD-corrected dissimilarities of each bin and those of environmental variables. The results revealed that several environmental factors, including total salinity, organic matter, and others, were significantly correlated with the community composition of these bins (Figure 8). Specifically, Bin1 was significantly associated with average plant height and top height; Bin3 was correlated with pH and total potassium; Bin14 showed significant correlations with available phosphorus and underground biomass; Bin19 was significantly associated with total nitrogen, available phosphorus, sucrase, protease, and non-capillary porosity; and Bin27 was linked to total potassium and average height.

## 4 Discussion

### 4.1 Plant-mediated soil modifications and their effects on rhizosphere fungal diversity and community composition

Our results support Hypothesis i, as PERMANOVA analysis confirmed significant differences in fungal community composition among plant types (*R*^2^ = 0.85, *p* = 0.02). This study found that rhizosphere fungal communities of different vegetation types showed significant differences in diversity and composition, similar to previous conclusions about degraded wetlands (Yan et al., 2024). The influence of plant types on fungal communities is mainly achieved through two levels of mechanisms: direct biochemical regulation and indirect environmental modification. In terms of direct mechanisms, plants regulate the composition and function of rhizosphere microorganisms by releasing organic acids, sugars, amino acids, and various secondary metabolites (Zhalnina et al., 2018; Wu et al., 2024). Meanwhile, root systems provide physical attachment sites and influence microenvironmental factors such as water retention, oxygen diffusion, and nutrient transport. These changes directly affect key physiological processes such as fungal hyphal growth and spore germination (Bonfante and Genre, 2010; Nazari et al., 2021). In this study, although detailed plant functional traits such as root architecture or exudate profiles were not directly measured, analyses based on plant type, biomass, and height still suggest potential trait-mediated effects on environmental modification. The comprehensive assessment of 20 soil and plant indicators implied that vegetation may indirectly influence fungal community assembly by altering soil structure and resource availability. Cluster analysis further divided the plant types into two groups (type 1, 3, 5 vs. type 2, 4, 6, 7) and revealed systematic environmental differences, suggesting that vegetation differences may affect fungal communities through multidimensional environmental filtering. From a functional perspective, these environmental differences may reflect general differences in growth strategies and biomass allocation. For example, high-biomass species (e.g., *A. donax* in type 2, 4, 6, 7) were associated with higher soil porosity and water retention, conditions that potentially enhance fungal colonization opportunities and strengthen deterministic assembly processes (Wardle et al., 2004; Freschet et al., 2021). In contrast, low-biomass species (type 1, 3, 5) may provide fewer belowground inputs, potentially restricting fungal dispersal and thereby amplifying dispersal limitation effects. While these interpretations remain tentative due to the limited trait measurements in this study, they point to a plausible link between plant growth characteristics and fungal assembly processes, which merits further targeted investigation.

This multi-level environmental regulation mechanism explains why different plants can selectively enrich specific fungal taxa. The community distribution patterns revealed by ternary diagram analysis reflect the specificity of plant-fungal interactions, fundamentally rooted in plants creating unique microhabitat conditions through differentiated growth strategies and resource allocation patterns. The functional performance of dominant fungal groups such as Sordariomycetes and Glomeromycetes in CWs is also closely related to this environmental differentiation. The former’s saprophytic function adapts to organic-rich environments, while the latter’s mycorrhizal function plays an important role in nutrient-limited environments (Tedersoo et al., 2014; Zhang et al., 2021; Hoosein et al., 2023). The independent response patterns exhibited by soil chemical, physical, and plant traits further indicate that fungal communities face the combined effects of multiple environmental filters. This multidimensional environmental control mechanism may be an important driving factor for the complexity and stability of fungal communities in constructed wetland systems.

### 4.2 Dominant mechanisms of rhizosphere fungal community assembly processes

The iCAMP analysis strongly supported Hypothesis ii, revealing dispersal limitation as the dominant mechanism (57.13%), a finding with important ecological significance. The dominance of dispersal limitation indicates that the spatial distribution of fungal communities is primarily controlled by biogeographical processes rather than direct screening by local environmental conditions, emphasizing the importance of spatial factors in microbial community construction. Similar studies have shown that land use changes cause multiple variations in rhizosphere fungal community assembly. For example, reclamation of abandoned farmland led to fungal community assembly shifting toward stochastic processes (Cao et al., 2024), and after Brazilian rainforest conversion to pasture, dispersal limitation contributed 55% to fungal β-diversity variation (Liu et al., 2023). Other studies have shown that reclamation of restored mining areas led to deterministic processes dominating fungal community assembly (Li et al., 2024), and after 30 years of converting farmland back to forest, fungal communities were dominated by strong environmental filtering effects (Chen et al., 2024; Yang et al., 2023). These seemingly contradictory results can actually be unified through differences in plant functional traits: the trait composition of plant communities (such as biomass and root depth) in different restoration scenarios determines the relative balance between deterministic and stochastic processes (Dini-Andreote et al., 2015; Tripathi et al., 2018). Compared to natural wetlands, constructed wetland systems typically have smaller spatial scales and younger ecosystem ages, which may explain the enhanced dispersal limitation effects. In our 3-year-established system, fungal communities may still be in the early colonization stage, where the influence of local dispersal and stochastic colonization events on community structure may exceed the role of environmental screening (Lan et al., 2022; Liu et al., 2023). Furthermore, physical barriers in CWs, agricultural patch fragmentation isolating fungal migration pathways, and limited connectivity may further exacerbate dispersal limitation effects (Jiao et al., 2021; Yu et al., 2022).

### 4.3 Regulatory mechanisms of environmental factors on specific fungal groups

Distance-based redundancy analysis confirmed available phosphorus as the primary environmental driver (marginal contribution = 0.137), supporting the second part of Hypothesis ii. Although the overall assembly process of fungal communities was dominated by stochastic mechanisms, iCAMP analysis results show that some functional groups (bins) are significantly influenced by environmental filtering in their community construction. Specifically, deterministic processes (mainly heterogeneous selection) contribute 20.53% to fungal community construction, indicating that specific environmental conditions still play a non-negligible role in screening dominant fungal groups. The iCAMP method identifies the microscopic mechanisms behind community construction by dividing communities into multiple ecological functional bins and quantifying the dominance of ecological processes experienced by each bin. Further analysis of bins identified as being dominated by non-neutral assembly processes (such as those with high HoS and HEs values) in the iCAMP analysis revealed that community differences in these bins were significantly correlated with multiple environmental factors, reflecting clear environmental filtering effects. For example, Bin3 and Bin31 were significantly regulated by pH, while Bin31 was also significantly associated with organic matter and total salinity, suggesting that these groups are highly sensitive to changes in soil chemical properties and may be driven by strong heterogeneous selection during the assembly process. Bin16 responded simultaneously to changes in organic matter and total salinity, emphasizing the combined effects of carbon source input and salt stress on the niche formation of this group. Furthermore, plant traits (such as belowground biomass and plant height) serve as important regulatory factors of the rhizosphere environment (Anderson et al., 2024) and also play roles in the construction of some bins. For example, Bin13 was significantly correlated with belowground biomass, and Bin1 was significantly correlated with average and maximum plant height, indicating that these groups may depend on microenvironments formed under specific plant growth states, further validating the close interactive relationship between plants and microorganisms. Nutrient supply further strengthened this environment-plant-microbe tripartite interaction mechanism. Bin17 was significantly correlated with available phosphorus, Bin19 with available nitrogen, and Bin14 with available phosphorus and belowground biomass, indicating that nutrient availability is an important factor determining niche differentiation of these fungal groups (Wang et al., 2024). It should be noted that although most bins showed some degree of stochastic driving, iCAMP results helped us identify those “key ecological groups” dominated by environmental filtering. This suggests that under the background of overall community assembly showing neutral trends, the ecological construction processes of specific groups still exhibit significant selectivity, thus revealing “macro-stochastic-micro-deterministic” structural characteristics at the microscopic scale.

### 4.4 Ecological significance of constructed wetland management

The results of this study have important guiding significance for the design and management of CWs. The importance of vegetation configuration strategies is reflected in the differential impacts of different plant types on fungal community diversity. Diversified vegetation configuration can not only maintain higher fungal diversity but also enhance system stability and resistance through functional complementarity effects (Wang et al., 2024). This study revealed that dispersal limitation dominates as the primary assembly mechanism, providing crucial scientific evidence for wetland construction timelines and spatial design. To effectively overcome dispersal barriers and accelerate ecosystem maturation, we propose the following specific strategies: (1) direct bio-inoculation using rhizosphere soil from mature wetlands during the initial construction phase, which can introduce diverse fungal propagules and effectively reduce colonization time. (2) adopt a “stepping-stone” vegetation configuration, establishing vegetation patches at 20–30 m intervals to facilitate fungal spatial dispersal through hyphal networks and spore movement. (3) create ecological connectivity corridors between wetland cells to enhance fungal migration capacity, which is particularly important in fragmented agricultural landscapes. These design principles based on dispersal limitation mechanisms can significantly improve the establishment efficiency of fungal communities in newly CWs.

Although stochastic processes dominate overall community assembly, homogeneous selection still accounts for a considerable proportion (20.53%), providing opportunities for targeted regulation of beneficial fungal groups. Our bin-level analysis revealed that specific fungal taxa exhibit predictable response patterns to environmental gradients. Based on this, managers can implement targeted environmental manipulation measures: enriching taxa preferring neutral conditions through pH adjustment (e.g., lime application), promoting saprophytic fungal development through organic matter enrichment (e.g., compost addition), and maintaining salt-sensitive taxa through salinity management. Furthermore, given that available phosphorus is the primary environmental driver (marginal contribution = 0.137), steer fungal communities toward desired functional compositions. Rhizosphere fungal communities have great potential as ecological health indicators. The sensitivity of fungal communities to environmental changes and their relatively stable taxonomic characteristics make them ideal indicators for assessing the ecological status of CWs. Specifically, monitoring the relative abundance of Sordariomycetes (saprophytic decomposers) and Glomeromycetes (Mycorrhizal symbionts) can serve as early-warning indicators of system health. Changes in the relative abundance of specific fungal groups can reflect changes in pollution levels, nutritional status, and ecosystem functions (Garcia-Gamboa et al., 2024), providing scientific basis for wetland management.

From a long-term management perspective, understanding fungal community assembly processes helps predict and regulate ecosystem development trajectories. By monitoring dynamic changes in key fungal groups, managers can timely adjust management strategies to maintain the healthy state of the ecosystem. Based on the results of this study, we propose a phased adaptive management framework: the establishment phase focuses on implementing bio-inoculation and spatial connectivity optimization to promote rapid fungal colonization; the development phase emphasizes monitoring key environmental drivers and adjusting substrate properties accordingly based on fungal community responses; the maturation phase shifts to maintenance mode, using fungal community indicators as the core for assessing system health and guiding precise interventions. This evidence-based phased management approach translates mechanistic understanding of fungal community assembly into actionable practical protocols, providing scientific guidance for wetland restoration practitioners to achieve desired ecological outcomes.

## 5 Conclusion

This study investigates the environmental factors influencing rhizosphere fungal communities in CWs with varying vegetation types. The analysis highlighted the significant roles of plant species identity, soil physicochemical properties, and plant biomass characteristics in shaping the diversity patterns and community assembly of these fungal communities. Our findings revealed distinct fungal community structures across different vegetation types. Both deterministic and stochastic processes were found to influence the establishment of these communities, with stochastic processes having a more dominant role. Specifically, among the stochastic processes, dispersal limitation emerged as the most significant factor (57.13%), followed by homogeneous selection (20.53%) and drift (19.91%). These patterns are likely driven by spatial constraints and the limited dispersal capacity of fungi. The partial Mantel tests further demonstrated the pivotal roles of plant species identity, soil physicochemical properties (such as salinity, organic matter, pH, and nutrient availability), and plant biomass in fungal community composition. Additionally, our findings emphasize the selective enrichment of certain fungal lineages by different plant types, underscoring the importance of plant-fungal interactions in wetland ecosystem functioning. This study contributes to understanding the assembly mechanisms and diversity patterns of rhizosphere fungal communities in CWs. The insights gained can inform future strategies for vegetation selection and microbial manipulation in polluted wetland ecosystem restoration projects.

## Data Availability

The original contributions presented in the study are publicly available. This data can be found here: NCBI, accession number PRJNA1373373.

## Footnotes

1
https://qiime2.org
